# Quality of life assessment septoplasty in patients with nasal obstruction

**DOI:** 10.1590/S1808-86942012000300011

**Published:** 2015-10-14

**Authors:** Thiago Freire Pinto Bezerra, Michael G. Stewart, Marco Aurelio Fornazieri, Renata Ribeiro de Mendonca Pilan, Fabio de Rezende Pinna, Francini Grecco de Melo Padua, Richard Louis Voegels

**Affiliations:** a PhD student – FMUSP (University of São Paulo Medical School) (ENT Physician).; b MD, MPH (Professor and Chairman, Department of Otolaryngology - Head & Neck Surgery).; c ENT Physician (Rhinology Fellow - FMUSP).; d PhD student – FMUSP (ENT Physician).; e PhD (Attending physician – HCFMUSP – FMUSP University Hospital).; f PhD (ENT Physician).; g Senior Associate Professor (Chairman of Rhinology - HCFMUSP). Faculdade de Medicina da Universidade de São Paulo. Associação Brasileira de Otorrinolaringologia e Cirurgia Cérvico-Facial.

**Keywords:** nasal obstruction, nasal septum, quality of life

## Abstract

Naasal obstruction is a common complaint in the population. When caused by a deviated nasal septum, septoplasty is the procedure of choice for treating these patients. NOSE is a tool for assessing the disease-specific quality of life related to nasal obstruction.

**Aim:**

To assess the impact of septoplasty on patients with nasal obstruction secondary to deviated nasal septum based on the disease-specific quality-of-life questionnaire. Design: Prospective.

**Methods:**

Patients undergoing septoplasty with/ without turbinectomy after no clinical improvement with medical treatment were assessed by the NOSE questionnaire before and 3 months after surgery. We evaluated the surgical improvement based on total score, the magnitude of the surgery in the disease-specific quality of life and the correlation between the preoperative score and postoperatively improvement.

**Results:**

Fourty-six patients were included in the study. There was a statistically significant improvement in the preoperative NOSE score (md = 75, IQR = 26) and after three months (md = 10, IQR = 20) (*p* < 0.001.T-Wilcoxon). The standardized response mean was 3.07. We found a strong correlation between the preoperative score in the NOSE questionnaire and improvements in the postoperative period (r = -0.789, *p* < 0.001, Spearman). No difference was found in improvement scores by gender. (*p* = 0.668, U-Mann-Whitney).

**Conclusion:**

Septoplasty resulted in a statistically significant improvement in the disease-specific QOL questionnaire.

## INTRODUCTION

Nasal obstruction is the feeling of blockage or insufficient air flow through the nose, and it can impact significantly on the individual's Quality of Life (QL). Its prevalence is 26.7% in urban centers^1^. Many are the causes for nasal obstruction, such as: rhinitis, adenoid hypertrophy, turbinate hypertrophy and sinonasal polyps. Nasal septum deviation is a very common cause of nasal obstruction, of simple diagnosis and ultimate treatment is based on septoplasty.

There is a tool used for the objective assessment of nasal obstruction: the NOSE^2^ questionnaire. After numerous studies published about the treatment of this complaint, with non-validated questionnaires^3^, ^4^, ^5^, Stewart et al. published the “Nasal Obstruction Symptom Evaluation Scale” in 2004^2^. Questionnaire validation to assess the disease-specific QL is an important means used to assess disease impact and patient treatment. In 2010, we published the transcultural and validation processes of this QL questionnaire for Brazil in an international journal^6^, with an established methodology concerning validation processes^7^ used for other QL questionnaires published for our language^8^. It is essential to have this stage prior to the use of a QL questionnaire developed in another language, because we need to culturally adapt it and validate it, instead of having a simple translation of it. This was the first otolaryngology validation in Brazil to use this international detailed and consolidated methodology, with the participation of the author of the original questionnaire^2^. This methodology avoided possible biases that could have happened should we have applied a simple translation.

There are some national studies which assessed the efficacy of nasal surgery to treat nasal obstruction; however, none of them used an instrument to evaluate disease-specific QL issues associated with nasal obstruction^9^, ^10^. This is the first Brazilian publication to use the validated version of the NOSE questionnaire.

The goal of the present paper is to assess the impact of septoplasty on the disease-specific quality of life of the patients with nasal obstruction secondary to a nasal septum deviation.

## PATIENTS AND METHODS

### Study design

This is a prospective study. All the patients agreed with the informed consent and signed the form which was authorized by the Ethics Committee of the Hospital (nº. 0521/08).

The primary goal of this study was to assess those patients with nasal obstruction who were submitted to septoplasty with or without turbinectomy, to treat nasal septum deviation, with or without hypertrophy of the inferior nasal conchae, respectively, as to improvements in the disease-specific QL, measured by the NOSE questionnaire three months after surgery.

Secondary goals were: to assess the correlation between the NOSE questionnaire pre-operative scores and the score variation after three months of the surgery; and to assess whether there is a differences in the disease-specific QL improvement according to gender.

### Patient Samples

The patients were consecutively recruited between June of 2008 and March of 2009. We used the following inclusion criteria: patients with chronic nasal obstruction caused by nasal septum deviation with or without nasal conchae hypertrophy; symptoms persisting for over 12 weeks; no response to the clinical treatment with topical steroids and anti-histaminic agents associated with nasal decongestants (only for patients with concurrent allergic rhinitis); surgical indication for septoplasty and age above 18 years.

We took off those patients with a history or diagnosis of sinonasal tumors; head and neck radiotherapy; septoplasty done with rhinoplasty or as an entry point to other sites; anterior nasal surgery; chronic rhinosinusitis (according to the criteria from EP3OS 2007)^11^; nasal septum perforation; craniofacial congenital bone changes; nasal trauma or fracture; adenoid hypertrophy; sarcoidosis or another Granulomatosis; asthma without clinical control; gestation.

### Treatment

Septoplasty is defined as an open surgery of the nasal septum with the goal of straightening it. The use of postoperative splints or nasal packing was not mandatory, nor evaluated.

The patients were submitted to septoplasty with or without inferior turbinectomy by resident ENT physicians from our department, according to the evaluation from the attending physician in indicating the surgery. The patients were independently evaluated by means of the NOSE questionnaire and the physician responsible for the patient was blind as to the preoperative and postoperative NOSE questionnaire scores.

The patients were medicated with postoperative isotonic nasal saline solution.

### Outcome measure

The outcome measure was the disease-specific QL measured by the NOSE questionnaire validated for the Portuguese language^6^ three months after surgery.

### Statistical analysis

The sample calculation was carried out considering an alpha lower than 5% and a beta lower than 20% for a 0.5 effect magnitude and 25% loss estimate, considering a total of 43 patients.

Data analysis was carried out using the SPSS 10.0 (SPSS Inc., Chicago, IL). The Kolgmorov-Smirnov test assessed the compliance concerning the distribution of values in the normal curve. The non-parametric Wilcoxon T test was utilized in order to compare the NOSE questionnaire scores before and three months after surgery. We also assessed surgery impact on the disease-specific QL. We assessed the correlation between the preoperative score and the postoperative improvement, calculated by the difference between the postoperative and preoperative scores, using the Spearman's correlation coefficient. Improvements in QL, as far as gender is concerned, were assessed by means of the Mann-Whitney U test. A p value lower than 5% was deemed significant.

## RESULTS

Forty-six patients with nasal obstruction secondary to nasal septum deviation with or without inferior nasal conchae hypertrophy were submitted to septoplasty and were included in the present study. Most of the patients were males [28/46 (69.1%)] with an age median (md) of 37.5 (interquartile interval (IIQ) = 17).

The most frequently preoperative answer to all the questions was: “A reasonably severe problem”. The preoperative median NOSE score was 75 (IIQ = 26). The most frequent postoperative answer to all the questions was “It is not a problem”. The postoperative NOSE median score was 10 (IIQ = 20).

**Figure 1:**
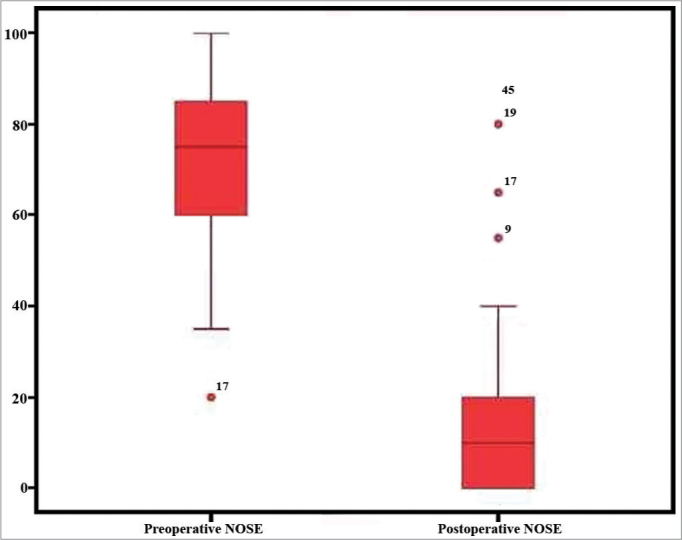
Pre and postoperative scores of the NOSE questionnaire (*p* < 0.001).

**Figure 2:**
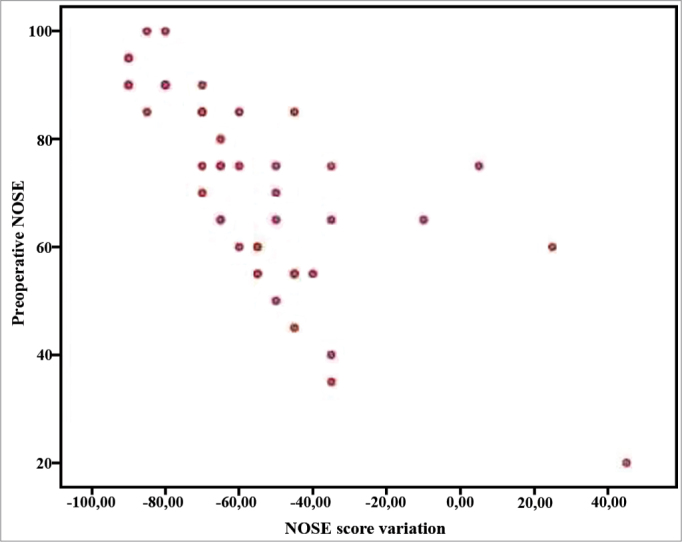
Correlation between the preoperative score in the NOSE questionnaire and the improvement in the score after three months (r = 0.789, *p* < 0.001).

There was a statistically significant improvement shown by the Wilcoxon T test between the preoperative NOSE questionnaire score (md = 75, IIQ = 26) and three months later (md = 10, IIQ = 20) (*p* < 0.001) (Figure 1). The surgery resulted in a standardized magnitude effect of 3.07. The Spearman coefficient showed a correlation of the NOSE questionnaire preoperative score and the postoperative score improvement (r = -0.789, *p* < 0.001) (Figure 2). The Mann-Whitney U test did not show statistically significant difference between the score improvements shown by each gender. (*p* = 0.668).

## DISCUSSION

This study confirms the hypothesis that septoplasty with or without turbinectomy for the treatment of adults with nasal obstruction secondary to nasal septum deviation, with or without inferior nasal conchae hypertrophy, respectively, results in improvements in the disease-specific QL measures using the NOSE questionnaire three months after the surgery.

The patients had statistically significant improvement in their NOSE questionnaire scores three months after surgery (md = 75, IIQ = 26 vs. md = 10, IIQ = 20) (p < 0.001) and a Strong statistically significant correlation between the improvement in postoperative score and the preoperative score in the NOSE questionnaire (r = -0.789, *p* < 0.001). The effect's magnitude was significant and three times higher than the standard deviation, indicating an important treatment effect.

We did not find differences between the genders in this sample of patients; despite the fact that quality of life studies published about other diseases showed a worsening in the quality of life among females^12^.

Limitations of this study were: non-randomized sample, to be carried out in a tertiary hospital, lack of a control group, not having validated the allergic rhinitis effect nor that of the turbinectomy on the patient's improvement. The use of a non-randomized sample, made up only of patients from our group of patients with surgical indication was inferior to the use of a randomized probabilistic sampling process, which could influence the result and its external validity. The difficulty in applying this type of sampling can be exemplified by the process of calling the patients from a waiting list one week before the surgery. The degree of patient discomfort at the time of the telephone call may lead the patient not to prioritize his surgery at that time because of private reasons, and we did not consider those patients who declined surgery at the time of calling.

If, on the one hand holding it in a tertiary hospital causes a patient selection bias, such fact brought about homogeneity as to the surgical technique utilized and the postoperative follow up. Although the surgeries were made by resident physicians, they were always supervised by preceptors, and the postoperative follows a unique service protocol.

The lack of a control group is justified for the fact that there was no efficient alternative treatment with proven efficacy to be administered after clinical treatment failure, and from the ethical stand point, a placebo treatment could not have been carried out since there is a widely accepted treatment for nasal septum deviation.

We recognized it would have been worth to assess the patients as to the presence of associated allergic rhinitis, and this could be validated in future studies. We did not assess the effects of associating turbinectomy because literature data from a previous study^13^ showed that such procedure did not influence the questionnaire's score variation. About the fact that we did not try to correlate the nasal obstruction severity by means of the degree of nasal septum deviation or the inferior nasal conchae hypertrophy, previous data have already shown the poor correlation between the anatomy and the questionnaire's score. It is important to stress that this fact is not exclusive to this questionnaire; self-answered QL questionnaires are a different construction from assessments carried out by the physician. There are other personal factors which impact the patient's perception about his problem. Patients with important symptoms and moderate nasal septum deviation could have a greater benefit from surgery than those with large deviations and mild symptoms. This could, at least partially, justify the fact that rhinometry has a good anatomical correlation, which is not always followed by a clinical correlation^14^.

It is important to highlight that the prospective design, the use of a validated questionnaire, patient assessment based on results, the reduction of the checking systematic error reduction and the lack of follow up losses were positive points in the present study.

Septoplasty is seen as an elective surgery, successful - most of the times. As Caldas Neto et al.^15^ reported: “it may harm the individuals QL, but in reality he will always have the option of living with the problem...”. Previous studies have analyzed and showed the efficacy of septoplasty in improving nasal obstruction and in promoting patient satisfaction^3^, ^5^, ^16^, ^17^, ^18^. Some of these studies were retrospective, through patient chart review which described only the opinion of the attending physician. Other studies used questionnaires by phone, non-validated questionnaires^5^, or even, validated questionnaires to assess QL as a whole, but not specific for nasal obstruction. Siegel et al.^4^ utilized a questionnaire validated for RS (rhinosinusitis) and showed an improvement in postoperative scores, but not of the global QL. Studies also utilized objective assessment by means of rhinometry, which true role is still very debatable^19^. It was only Stewart et al.^13^ who published an assessment of septoplasty efficacy using a specific QL for nasal obstruction, and so far, there were no Brazilian publications on the issue. During the bibliographic review, we were surprised to see that there were no studies published in Brazil about the efficacy of septoplasty to treat nasal obstruction secondary to nasal septum deviation, only based on assessing the turbinectomy^9^, ^10^.

There are other disease-specific QL questionnaires available to assess nasal complaints, but none of them is specific to assess the nasal obstruction only: the “Chronic Sinusitis Survey (CSS)”^20^, the “Rhinosinusitis Disability Index (RSDI)”^21^, the “Sino-Nasal Outcome Test (SNOT-20)”^22^, the “Rhino-conjunctivitis Quality of Life Questionnaire (RQLQ)”^23^, and the “Allergy Outcome Survey (AOS)”^24^. The CSS, the RSDI and the SNOT-20 were made to assess the chronic rhinosinusitis, just as the RQLQ and the AOS, for allergic rhino-conjunctivitis. Except for the SNOT-20, none of the others are validated for the Portuguese language.

Although there are questionnaires which have some relationship in showing the nasal obstruction, it is necessary to have a specific instrument for nasal obstruction^21^, ^23^, ^25^.

The NOSE questionnaire utilized to assess the specific QL associated with the nasal obstruction is a simple and fast questionnaire to answer. Its score varies between 0 and 100, with higher scores meaning greater nasal obstruction. Validated in English by Stewart et al.^2^; its transcultural adaptation and validation into Portuguese were carried out with the help of the author of the original questionnaire^6^. The goal of this recently published process was to make a tool available to assess the disease-specific QL of patients with nasal obstruction, maintaining its original meaning. A simple translation would not be adequate and could lead to an instrument which would not be equivalent to the original questionnaire, and this would limit response comparison between the populations^25^. The validation process enabled us to perform this observational prospective study in order to assess the septoplasty efficacy in improving the QL of patients. The correlation between the preoperative score and the magnitude of the postoperative response in the NOSE questionnaire confirmed what had already been shown in previous studies in which the NOSE13 was employed: those patients with a greater impact on their QL tend to have a greater improvement after the procedure.

## CONCLUSION

The patients with nasal septum deviation with or without turbinate hypertrophy submitted to septoplasty had improvements in their disease-specific QL for nasal obstruction assessed by means of the NOSE questionnaire. There was a correlation between a worse pre-op QL and a greater QL development after surgery.
